# A crystallization apparatus for temperature-controlled flow-cell dialysis with real-time visualization

**DOI:** 10.1107/S1600576716004635

**Published:** 2016-04-22

**Authors:** Niels Junius, Esko Oksanen, Maxime Terrien, Christophe Berzin, Jean-Luc Ferrer, Monika Budayova-Spano

**Affiliations:** a Université Grenoble Alpes, IBS, F-38044 Grenoble, France; b CNRS, IBS, F-38044 Grenoble, France; c CEA, IBS, F-38044 Grenoble, France

**Keywords:** optimization of crystal growth, phase diagrams, dialysis, temperature control, macromolecular crystallography

## Abstract

Many instrumentation developments in crystallization have concentrated on massive parallelization assays and reduction of sample volume per experiment to find initial crystallization conditions. Yet improving the size and diffraction quality of the crystals for diffraction studies often requires decoupling of crystal nucleation and growth. This in turn requires the control of variables such as precipitant and protein concentration, equilibration rate, and temperature, which are all difficult parameters to control in the existing setups. The success of the temperature-controlled batch method, originally developed to grow very large crystals for neutron crystallography, demonstrated that the rational optimization of crystal growth has potential in structural biology. A temperature-controlled dialysis button has been developed for our previous device, and a prototype of an integrated apparatus for the rational optimization of crystal growth by mapping and manipulating temperature–precipitant concentration phase diagrams has been constructed. The presented approach differs from the current paradigm, since it involves serial instead of parallel experiments, exploring multiple crystallization conditions with the same protein sample. The sample is not consumed in the experiment and the conditions can be changed in a reversible fashion, using dialysis with a flowing precipitant reservoir as well as precise temperature control. The control software allows visualization of the crystals, as well as control of the temperature and composition of the crystallization solution. The rational crystallization optimization strategies presented here allow tailoring of crystal size, morphology and diffraction quality, significantly reducing the time, effort and amount of expensive protein material required for structure determination.

## Abstract 2

An apparatus for the optimization of crystal growth using precise temperature control and dialysis combined with real-time visualization has been developed.

## 1. Introduction   

In X-ray protein crystallography, obtaining crystals of a protein is a delicate step, but it is considerably more laborious to improve these crystals up to a sufficient size and quality for diffraction. The availability of powerful radiation sources, fast detectors and cryocooling techniques has not removed the need for larger and better ordered crystals. Recording data from very small crystals requires significantly longer measurement times and, because of radiation damage, dozens (even hundreds) of crystals may be required in order to collect a complete set of data. The quality of data obtained is often lower than it could be with larger crystals, which may be crucial in experimental phasing, for example (Rice *et al.*, 2000 ▸).

Furthermore, a major challenge in fast (sub-millisecond) time-resolved X-ray crystallography where the reaction is initiated by light is the uniform initiation across the whole crystal volume (Levantino *et al.*, 2015 ▸). Varying activation fractions across a crystal, or a crystal population in serial femtosecond crystallography, makes accurate downstream scaling and data analysis to examine the time-dependent changes extremely difficult. One way to obtain more even photoexcitation is to have a very uniform population of crystals (in terms of size and morphology); the optimal crystal size for maximum scattering and maximum laser light penetration is ∼10–20 µm.

In neutron macromolecular crystallography where the available neutron sources are weak, compared to X-ray sources, the crystal volume required for a neutron data set is most often the factor limiting the more widespread use of this technique. If normal hydrogenated proteins are used, a minimum crystal size of at least 1 mm^3^ is necessary. If perdeuterated proteins are used the minimum crystal size can be as ‘little’ as approximately 0.15 mm^3^ (Blakeley, 2009 ▸). However, even with these large crystals, a single neutron diffraction data set can take several days or weeks to collect. For neutron crystallography to become more accessible to the structural biology community, the ability to control crystal size must become routine. This is particularly important in order to push the limits of neutron crystallography towards more challenging targets such as membrane proteins. Despite the knowledge gained in crystallization of model systems (Asherie, 2004 ▸; Vekilov, 2005 ▸), the principles are often difficult to implement in practice. Many proteins are not readily available in large quantities, which has driven crystallization methods towards small sample volumes often using vapour diffusion. One disadvantage of these methods is that the level of supersaturation is difficult to control effectively, especially as a function of time. Novel devices (Talreja *et al.*, 2010 ▸; Boudjemline *et al.*, 2011 ▸; Pusey, 2011 ▸; Meyer *et al.*, 2012 ▸; Abdallah *et al.*, 2013 ▸) have attempted to address this issue with somewhat different strategies, such as controlling the vapour pressure externally or using (micro)fluidics, dual polarization interferometry or fluorescence-based approaches.

Temperature control and dialysis are powerful tools for the separation of nucleation and growth. Dialysis allows the separation of molecules according to their size by a semi-permeable membrane whose pores are smaller than the macromolecular dimensions. These pores allow small mol­ecules, such as the solvent, salts and small metabolites, to diffuse through the membrane but block the passage of larger molecules.

**Figure 1:**
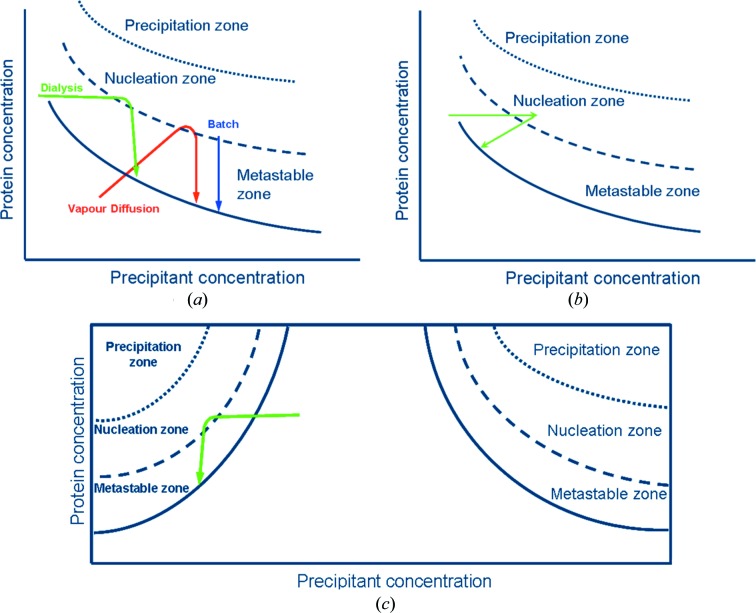
Schematic view of a phase diagram as a function of the concentration of precipitant, with the kinetic trajectories illustrated by arrows in the case of different dialysis crystallization modes: (*a*) comparison between vapour diffusion (red), batch (blue) and dialysis (green) salting-out experiments, (*b*) dialysis by changing the concentration of the precipitant during the crystallization experiment, and (*c*) a dialysis salting-in crystallization experiment (desalting) where the trajectory is in a different part of the phase diagram.

Dialysis as a crystallization technique involves diffusion and equilibration of precipitant molecules through a semi-permeable membrane as a means of slowly approaching the concentration at which the protein crystallizes (Fig. 1 ▸ *a*). Initially, the protein solution is contained within the dialysis chamber, which is then equilibrated against a precipitant solution through the semi-permeable membrane. Equilibration against the precipitant in the surrounding solvent slowly achieves supersaturation for the protein within the dialysis chamber, eventually resulting in crystallization. The precipitating solution can also be varied, simply by removing the initial precipitant solution and exchanging it with another (Fig. 1 ▸ *b*). The protein material can thus be reused until the correct conditions for nucleation and crystal growth are found. Dialysis can also be used to exploit the salting-in region of the phase diagram where solubility increases with increasing precipitant concentration (Fig. 1 ▸ *c*). Specifically, for a charge-neutral species (*i.e.* proteins at their pI) the salting-in effect dominates initially (protein solubility increases) and the addition of salts disrupts attractive protein–protein interactions. Then, further increase in (kosmotropic) salt concentration results in strengthening attractive protein–protein interactions as the salting-out effect begins to dominate (protein solubility decreases). The surface charge density of a protein could also dramatically change the above behaviour. At a pH close to the pI or for a large-size protein with a small number of either positive or negative net charges, where the surface charge density is low, only the monotonic salting-in behaviour could be observed because the charge neutralization process is less dramatic. On the other hand, when a protein has high surface charge density owing to either a small size or a large number of positive charges, the anions might not completely neutralize the positive charges even at molar concentration and therefore only a decrease in protein solubility can occur (Zhang, 2012 ▸). Accordingly, in the salting-in region the protein can be crystallized by lowering the precipitant concentration and forcing the protein out of solution (Fig. 1 ▸ *c*). Finally, dialysis allows the supersaturation to be varied in a reversible manner, so that crystals can be grown and dissolved as long as no irreversible precipitation or denaturation occurs.

Temperature is another useful variable in crystallization of biological macromolecules (Budayova-Spano *et al.*, 2007 ▸; Astier & Veesler, 2008 ▸; Leng & Salmon, 2009 ▸; Selimović *et al.*, 2010 ▸). Using precise temperature makes it possible to control the supersaturation reversibly. The full potential of temperature as a crystallization variable is often not realized as it is difficult to control precisely in typical crystallization setups.

We have previously developed an instrument for temperature-controlled batch crystallization (Budayova-Spano *et al.*, 2007 ▸), intended specifically for neutron protein crystallography. This instrument allowed the rational optimization of large crystal growth based on a phase diagram, although in many cases an explicit measurement of the solubility curve is actually not necessary.

We describe here a succession of evolutions of the previous instrument. Firstly, we have developed a temperature-controlled dialysis button: a modification to the existing device (Budayova-Spano *et al.*, 2007 ▸) that enables a temperature-controlled dialysis crystallization experiment. Secondly, we describe a new crystal growth apparatus with a temperature-controlled dialysis setup. It combines accurate temperature control with control of the composition of the crystallization solution (*e.g.* precipitant concentration, pH, additive) in an automated manner (Budayova-Spano, 2010 ▸). The apparatus described here allows sophisticated experiments to be performed, including systematic phase diagram investigation in multi-dimensional space using far less protein material than previously.

## 2. Methods   

### 2.1. Crystallization   

Chicken egg-white lysozyme was purchased from Sigma Aldrich as a lyophilized powder, dissolved in distilled water and filtered to obtain a solution with a final concentration of about 30 mg ml^−1^. Atk1, a kinase from *Agrobacterium tumefaciens* (F. Borel, S. Richard, F. Pojer, L. Jacquamet, T. Baiga, J. A. Ramsey, A. Iannello, M. Bowman, J. P. Noel & J.-L. Ferrer, unpublished data), was used with a final concentration of about 10 mg ml^−1^. The protein concentration was measured *via* the UV absorbance at 280 nm. All solutions were filtered through 0.22 µm Millipore filters. The cellulose membranes used in our experiments were the standard RC membrane Spectra/Por (http://www.spectrumlabs.com/) with a molecular weight cutoff of 6–8 kDa. Before the start of the experiment, the crystallization mixtures were centrifuged and filtered to remove all solid particles (precipitate, dust or nuclei).

Crystallization condition for dialysis experiment in the case of lysozyme: 15 µl of protein solution was placed in the crystallization chamber of the temperature-controlled flowing dialysis setup. The initial conditions of the reservoir solution were 0.75 and 0.9 *M* NaCl, 100 m*M* Na acetate pH 4, and the initial temperatures were 291 and 295 K (see §3).

Crystallization condition for dialysis in the case of Atk1: 25 µl of protein solution was placed in the crystallization chamber of the temperature-controlled dialysis buttons. The initial conditions of the reservoir solution were 18, 19, 20 and 21% of PEG 8000, MgCl_2_ 5 m*M*, adenosine triphosphate 5 mM, 100 m*M* Na citrate pH 6.

In the case of Atk1 and lysozyme crystals’ characterization with X-rays, temperature-controlled dialysis buttons with a volume of 25 µl of protein solution, rather than a flowing dialysis setup, were used in parallel under the previously described initial conditions of the reservoir solutions at constant temperature (293 K).

Finally, regarding the vapour diffusion crystallization experiment in the case of lysozyme, carried out also at 293 K, drops composed of a mixture of a volume of protein sample with an equal volume of reservoir solution were placed in vapour equilibration with a liquid reservoir solution described previously (Table 1 ▸). In order to achieve nucleation by vapour diffusion in the case of Atk1, the reservoir solution used must have a higher PEG 8000 concentration (25%) compared to that which was used in dialysis (18–21%) (Table 2 ▸).

### 2.2. Temperature-controlled dialysis button   

**Figure 2:**
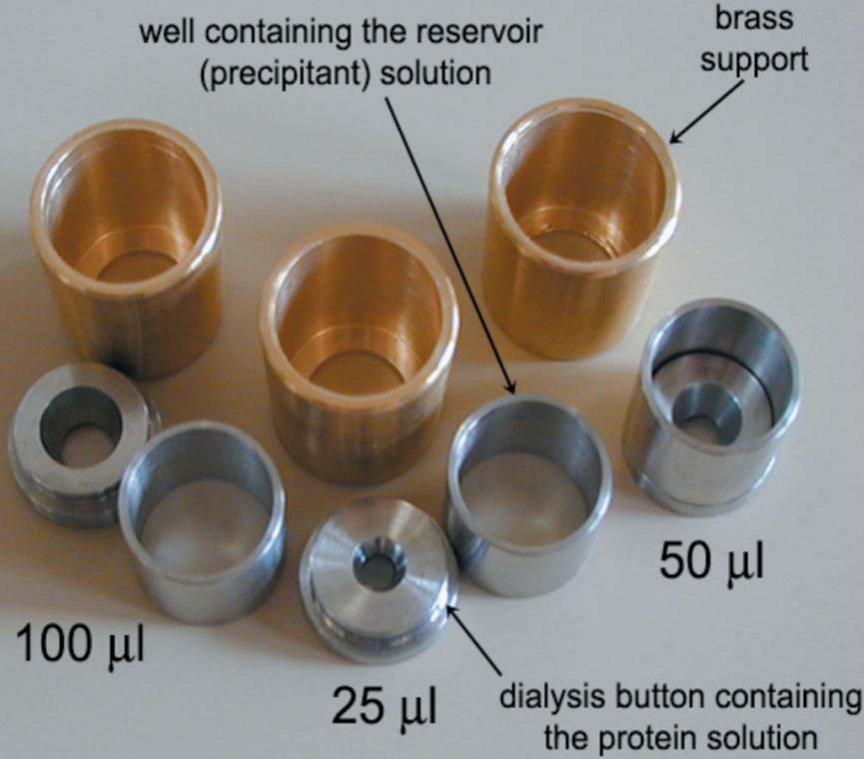
Photograph of various temperature-controlled dialysis buttons.

The temperature-controlled dialysis button allows our previous instrument (Budayova-Spano *et al.*, 2007 ▸) to be used for dialysis in addition to batch crystallization. The protein solution is poured into a specially designed stainless steel dialysis button with a polycarbonate window at the bottom (Fig. 2 ▸). It is separated from the precipitant solution by a dialysis membrane (of the appropriate molecular weight cutoff and other characteristics). The dialysis membrane is placed over the top of the dialysis chamber containing the sample (a variety of sizes of 25–200 µl are available) and is held in place with an elastic ring in a groove.

A stainless steel reservoir well is then placed over the dialysis button in order to ensure the water tightness. The well contains up to 1 ml of the precipitant solution. The reservoir well is sealed with vacuum grease and a glass cover slide or with sealing tape. The dialysis setup is attached to a brass support incorporating one or several wells of the temperature-controlled crystal growth apparatus (Budayova-Spano *et al.*, 2007 ▸). With this setup, control of the temperature of the crystallization experiment is performed in an automated way, while the chemical composition of the reservoir solution must be changed manually by the user.

To recover the crystals, the reservoir well containing the precipitant solution is removed and a surgical blade is used to make an incision in the membrane around the outer circumference of the sample chamber opening for crystal harvesting.

### 2.3. Temperature-controlled flowing reservoir dialysis setup   

**Figure 3:**
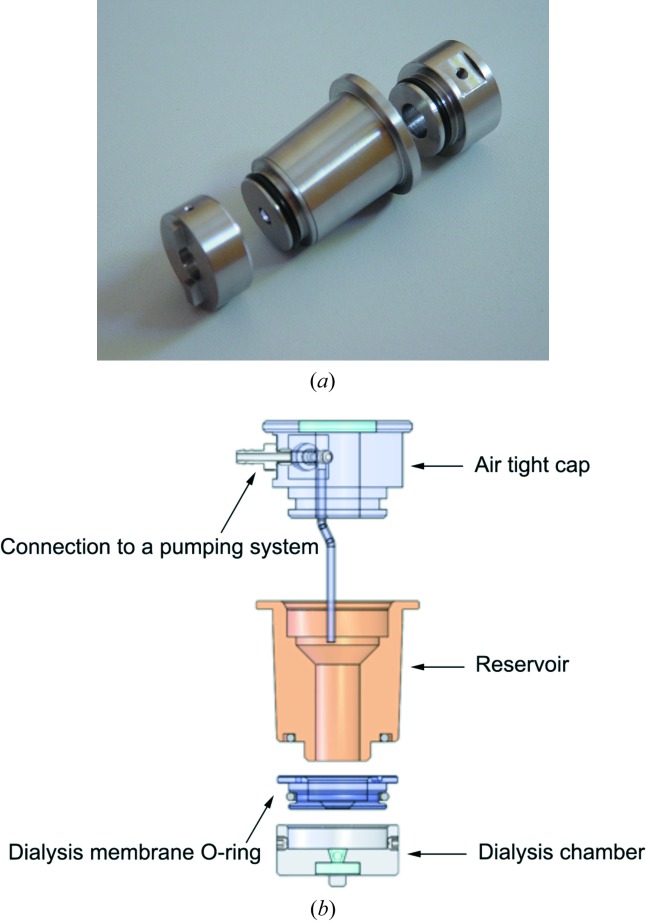
(*a*) Photograph of the temperature-controlled flowing reservoir dialysis setup. (*b*) Schematic view of the temperature-controlled flowing reservoir dialysis setup.

In this new version, the dialysis button is replaced by a new fluidic system in order to exchange the chemical composition in an automated way during the crystallization experiment. Now, the temperature-controlled flowing reservoir dialysis setup consists of three main parts (Fig. 3 ▸). The crystallization chamber, named hereafter the dialysis chamber, with a volume of 15 µl, houses the protein solution. It has a transparent polycarbonate window at the bottom. The dialysis membrane that separates the dialysis chamber from the reservoir solution is attached to an overchamber that allows the assembly composed of the dialysis chamber and the overchamber to be removed from the dialysis flow-cell setup for crystal mounting without removing the dialysis membrane. The reservoir chamber, which contains crystallization agents, additives and buffers, is placed on top of this assembly. The reservoir is covered by a plug equipped with an optical window to allow top illumination of the dialysis chamber. The reservoir chamber is connected to a pump, thus functioning as a continuous flow cell.

The user may adjust the composition of the reservoir solution and hence the crystallization condition. Precise control of the concentration of all components of the flowing reservoir (*e.g.* precipitant concentration) is performed using a pressure-driven flow control system.

**Figure 4:**
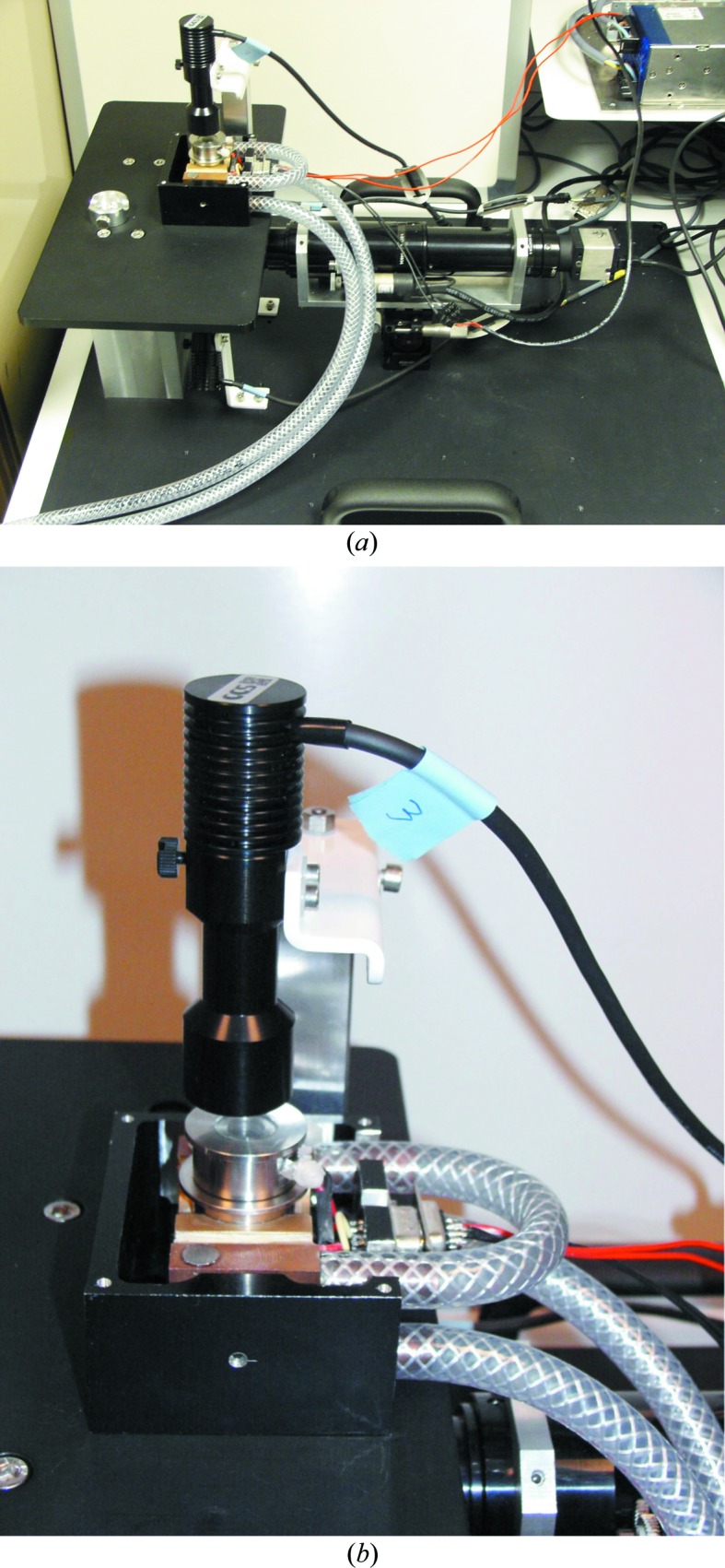
Photograph of the new apparatus for temperature-controlled flowing reservoir dialysis (*a*), with a detailed view of the temperature-controlled flowing reservoir dialysis setup within the apparatus (*b*).

The flowing reservoir dialysis setup is inserted into a brass support in thermal contact with Peltier elements of a crystal-growth apparatus that is incorporated on the microscope table (Fig. 4 ▸ *b*). The crystallization chamber is viewed from below by an inverted microscope (Optics Peter, Lentilly, France) with a digital video camera, a motorized 12× zoom and XYZ translation stages (Fig. 4 ▸ *a*). Illumination is provided by three light emitting diodes (one direct light source and two back light sources).

A proportional-integral-derivative electronic temperature controller (Laird Technologies PR-59) allows a temperature range of 233.0–353.0 ± 0.1 K. The cooling of the Peltier elements with a chiller (Julabo) improves temperature control. A dry air flow prevents condensation of air humidity, especially at low temperatures. A Fluigent (FASTABTM-based technology) pressure-driven flow controller allows one to control independently up to four channels of a fluidic system.

**Figure 5:**
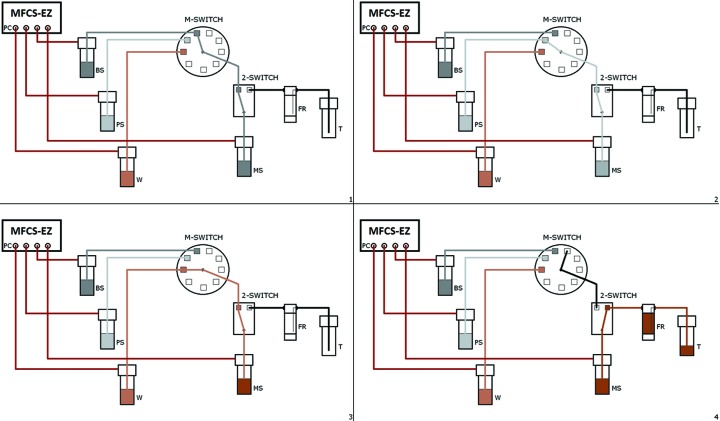
Multichannel fluidic system applications: principle of the fluid mixing system. Pressure channels (PC) allow us to pressurize containers to push the solution from the container into the tubing systems. The first stock solution from the buffer stock (BS) container is injected into the mixing tube solution (MS) *via* the multiple-switch (M-SWITCH) and the 2-SWITCH (step 1). Then the channel of the M-SWITCH is changed using a switchboard which allows the communication of the M-SWITCH and 2-SWITCH. The second solution from precipitant stock (PS) is then injected in the same manner as the first solution (step 2). Finally the M-SWITCH channel is modified again to complete the mixing of the solution with deionized water (W) (step 3). When the mixed solution is ready to use for crystallization the 2-SWITCH channel is modified to allow the filling up of the flowing reservoir (FR). At the end of the flowing process a trash container (T) is used to collect the solution already used for crystallization (step 4). If all of the mixed solution has been used a new one can be prepared in the same way. If not the tube can easily be removed and replaced by a new container.

**Figure 6:**
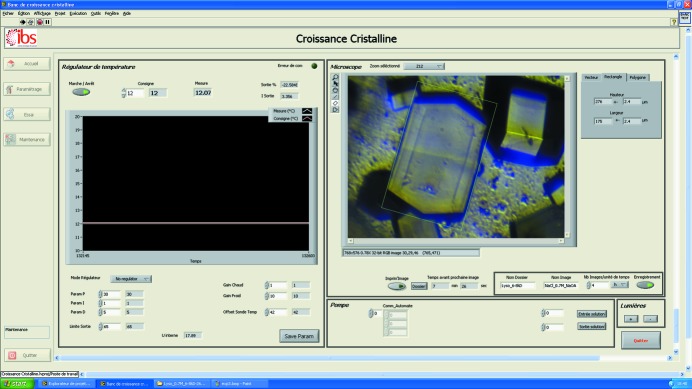
A graphical user interface in *LabVIEW* for system setup, viewing, monitoring, treating images, recording film sequences, regulating the temperature, pumping the solutions and measuring crystals.

This pressure-driven pumping system is controlled by dedicated software that allows the user to specify the nature and the concentration of the required crystallization agents, additives and buffers, and therefore to produce suitable mixtures from the different stock solutions for the reservoir solution of the flow-cell dialysis cell. Fig. 5 ▸ illustrates how this mixing system works. The control software is written with *LabVIEW* (http://www.ni.com/labview/) and includes a graphical user interface (Fig. 6 ▸) for visualization and measurement of crystals and image acquisition, processing and storage, as well as control of each parameter (temperature control, illumination, pumping the solutions and calculating the concentration of different constituents of the crystallization solution).

The configuration of the flowing reservoir dialysis setup in three portions enables recovery of the crystals at the end of the experiment. The crystallization solution is removed, for example using the pressure-driven pump, and the reservoir chamber is unscrewed. The overchamber with the dialysis membrane remains attached to the dialysis chamber so as to preserve the crystals against any mechanical damage or drying. The crystals can then be harvested as described above (§2.2) using the integrated microscope.

### 2.4. Crystal characterization with X-rays   

The diffraction data were collected at the FIP-BM30A beamline at the European Synchrotron Radiation Facility (ESRF). One hundred and eighty diffraction images were collected from each crystal using an X-ray wavelength of 0.979 Å. Comparable exposure times were used for all crystals. The data were indexed and integrated using the *XDS* software (Kabsch, 2010 ▸).

## 3. Results   

The temperature-controlled flowing reservoir dialysis setup system is an integrated automated version of the existing instrument for temperature-controlled crystallization, where the crystallization batch has been replaced with dialysis. It consists of a crystal growth bench that, in addition to accurate temperature control, allows the chemical composition of the crystallization solution to be varied in an automated manner thanks to a dialysis cell equipped with a flowing reservoir. The current sample volume is 15 µl per experiment, but various volumes from a few to a few tens of microlitres are feasible. With this system, it is possible to optimize the kinetic path though the phase diagram, which controls the nucleation and growth of the crystals, and thus their number, size and morphology.

**Figure 7:**
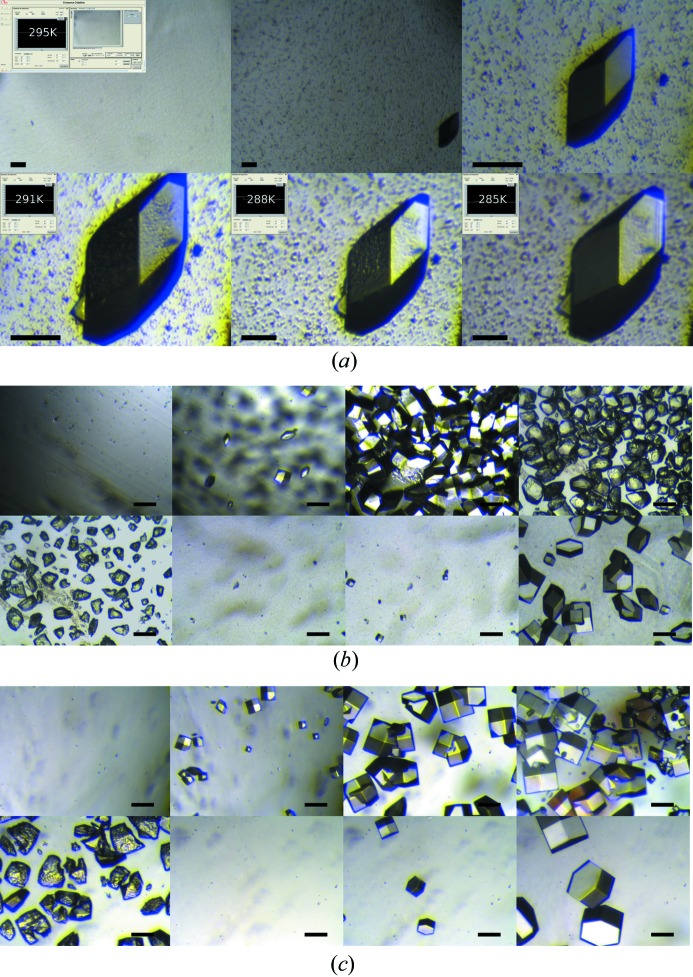
The dialysis crystallization experiment with variation of the temperature using the integrated apparatus for optimization of crystallization in the case of chicken egg-white lysozyme. The scale represents 100 µm; images are ordered from left to right. (*a*) The first three images show the initial growth at 295 K with two different zooms. Then the crystal is shown at the beginning of temperature change to 291 and 288 K (zoom is not the same to fit the crystal size). The last image shows the crystal at the end of the crystal growth process at 285 K. (*b*) The first three images show the initial nucleation and crystal growth at 291 K. Then the temperature is increased to 313 K until total dissolution of all the crystals (next three images). The two last images show the second nucleation event and related crystal growth at 295 K. The precipitant concentration was kept constant at 0.9 *M* NaCl during the entire experiment. (*c*) The first four images show the initial growth at 0.9 *M* NaCl. Once the precipitant concentration has been lowered gradually to 0 *M* NaCl, replacing it with only Na acetate buffer at 0.1 *M* pH 4, successive dissolution of all the crystals is observed (next two images). The two last images show the second nucleation event and related crystal growth at 0.75 *M* NaCl. The temperature was kept constant at 291 K during the entire experiment.

We performed a proof of principle experiment with chicken egg-white lysozyme to demonstrate the control of nucleation and crystal growth (Fig. 7 ▸). Fig. 7 ▸(*a*) demonstrates the controlled large crystal growth of a lysozyme single crystal. In this experiment, the initial condition was 0.75 *M* NaCl, 100 m*M* Na acetate pH 4 at 295 K. The estimated time to reach an equilibrium between the reservoir and the dialysis chamber is 90 min. After this time the supersaturation in the dialysis chamber is low enough that a few nuclei are formed, and the first visible nuclei appear 22 h after equilibration. Then the crystal continues to grow until no more macroscopic growth is observed, which is in agreement with the previously established procedure for large crystal growth in batch (Budayova-Spano *et al.*, 2007 ▸). After three days the temperature was decreased to 291 K to restart the crystal growth. One day later the temperature was decreased to 288 K, and four days later when the crystal no longer showed visible growth the temperature was decreased again to 285 K until the crystal exceeded the size of 500 µm in the longest dimension. Ten days after the start of the experiment, the overall crystal growth process was intentionally interrupted in order to demonstrate how to obtain a large single crystal of a model protein with a volume approaching 0.1 mm^3^. This is the volume typically needed in neutron protein crystallography when a protein is perdeuterated.

We then performed two experiments to demonstrate the reversibility of the dialysis experiment for nucleation, crystal growth, dissolution and re-growth of crystals using temperature and precipitant variations, respectively. The initial crystallization condition was 0.9 *M* NaCl, 100 m*M* Na acetate pH 4 at 291 K in order to induce significant nucleation. After less than 60 min many crystals appeared in the dialysis chamber, and these grew for 3 h at 291 K to medium-sized crystals.

In the first experiment a temperature gradient was used (Fig. 7 ▸ *b*) while the concentration of the precipitant was kept constant. The temperature was increased gradually to 313 K over 20 min to start the dissolution process. The dissolution of the crystals was complete in 1 h. The temperature was then rapidly decreased to 295 K (typically in a few minutes) to induce less nucleation than at the beginning of the experiment. This is consistent with the fact that the solubility of the lysozyme crystals increases with temperature. As the dialysis chamber was already at equilibrium with respect to the precipitant concentration, the second nucleation event appeared faster than the first one. Thus, in this second nucleation event, after 18 min, the first nuclei appeared and with a lower density, as expected from the theoretical phase diagram.

In the second experiment a concentration gradient of precipitant (Fig. 7 ▸ *c*) was used at constant temperature (291 K). Here the dissolution process of lysozyme crystals is due to the gradient of the NaCl concentration, which was decreased gradually to 0 *M* so that finally only the Na acetate buffer at 0.1 *M* pH 4 was circulating in the reservoir. The dissolution did not appear immediately after the concentration change because of the equilibration time required between the reservoir and the dialysis chamber. The crystals continued to grow for a short period of time, and after 60 min the smallest nuclei began to dissolve. The dissolution was complete in 2 h. Finally the precipitant concentration was increased again to a reservoir NaCl concentration of 0.75 *M*. As expected from the theoretical phase diagram, nucleation was observed after an equilibration time of 90 min, but with a lower density than originally.

To verify the diffraction quality of the crystals grown in the temperature-controlled dialysis setup we collected X-ray diffraction data from three crystals of lysozyme and six crystals of Atk1 grown in parallel in temperature-controlled dialysis buttons and compared them with three crystals of comparable size and appearance of lysozyme and Atk1, each grown in hanging drops in a vapour diffusion setup. The crystals grown in our temperature-controlled dialysis setup clearly have better diffraction quality than the crystals grown by vapour diffusion for both lysozyme (Table 1 ▸) and Atk1 (Table 2 ▸). All of the crystals we tested had a lower mosaicity and a higher diffraction limit than the reference crystals grown by vapour diffusion.

## 4. Discussion   

Different empirical approaches have been developed to produce crystals, based on screening and optimization (Caffrey, 2009 ▸; Garcia-Caballero *et al.*, 2011 ▸; Gourdon *et al.*, 2011 ▸; Luft *et al.*, 2014 ▸). The knowledge of the phase diagram and the specific control of the crystallization parameters such as the temperature and the concentration of crystallization agents and/or additives will allow the number of crystals and their macroscopic defects to be reduced, as well as the selection of the nucleation and/or growth of the desired phase. Even when the precise position of the solubility curve on the phase diagram is not experimentally known, the ability to control the crystallization parameters in a reversible manner together with real-time observation of the crystals allows the phase diagram to be explored in a qualitative way.

The devices described here allow multiple crystallization conditions to be explored in a systematic way with the same biological sample. The sample is not consumed in the experiment and, as long as it is not damaged (*e.g.* denatured), the conditions can be explored reversibly. Tailoring of crystal number, size, phase and diffraction quality reduces the time, amount of protein material and effort required for structure determination. The approach differs from the current paradigm in performing serial instead of parallel experiments.

We expect the described devices to be useful in monitoring and controlling the crystallization processes that allow production of crystals of specific sizes and morphology optimized for different downstream structure determination approaches. This will be beneficial for free-electron laser and synchrotron serial crystallography experiments which require large numbers of small crystals (<50 µm) with a very uniform population of crystals (in terms of size and morphology), and for neutron studies that require large single crystals to provide sufficient scattering volumes (0.1–1.0 mm^3^, depending on the deuteration approach). Several neutron crystallography targets are being investigated with the present system, and the positive results already obtained together with the related crystallization protocols will be published in the near future. Our results indicate that the control of crystal growth does not compromise the diffraction quality, and rather improves it (Tables 1 ▸ and 2 ▸), but this effect remains to be demonstrated on a larger number of systems.

In addition, the setup is applicable to many other objectives such as studying salting-in and salting-out effects in crystallization, gentle addition and exchange of buffers, additives, ligands, heavy metals, detergents or cryoprotectants, and cross-linking and dehydration or isotope exchange (H_2_O *versus* D_2_O) of crystals for diffraction purposes in X-ray or neutron protein crystallography, respectively.

## Figures and Tables

**Table 1:** Summary of the diffraction characterization of lysozyme crystals grown by hanging drop vapour diffusion and the temperature controlled dialysis method

Crystallization method	Hanging drop	Hanging drop	Hanging drop	Dialysis	Dialysis	Dialysis
NaCl (*M*)	0.9	0.9	0.75	0.9	0.75	0.75
Largest dimension (µm)	200	100	300	200	300	100
Resolution† (Å)	50–1.36 (1.44–1.36)	50–1.30 (1.38–1.30)	50–1.32 (1.40–1.32)	50–1.25 (1.33–1.25)	50–1.16 (1.23–1.16)	50–1.15 (1.22–1.15)
Mosaicity (°)	0.332	0.235	0.328	0.220	0.128	0.136
*I*/σ(*I*)†	27.19 (2.04)	25.33 (2.12)	20.30 (2.11)	25.28 (2.05)	29.19 (2.24)	25.40 (2.02)
CC(1/2)‡	100.0 (73.0)	100.0 (81.8)	100.0 (75.2)	100.0 (78.8)	100.0 (78.4)	100.0 (69.8)

† Highest resolution shell in parentheses.

‡ Karplus & Diederichs (2012 ▸).

**Table 2:** Summary of the diffraction characterization of Atk1 crystals grown by hanging drop vapour diffusion and the temperature-controlled dialysis method

Crystallization method	Hanging drop	Hanging drop	Hanging drop	Dialysis	Dialysis	Dialysis	Dialysis	Dialysis	Dialysis
PEG %	25	25	25	21	20	19	19	18	18
Largest dimension (µm)	100	200	300	150	100	250	400	200	400
Resolution† (Å)	50–1.85 (1.96–1.85)	50–1.79 (1.90–1.79)	50–1.87 (1.99–1.87)	50–1.61 (1.71–1.61)	50–1.67 (1.77–1.67)	50–1.55 (1.64–1.55)	50–1.34 (1.42–1.34)	50–1.41 (1.49–1.41)	50–1.22 (1.29–1.22)
Mosaicity (°)	0.354	0.397	0.578	0.270	0.223	0.187	0.259	0.205	0.259
*I*/σ(*I*)†	14.54 (2.03)	13.64 (2.07)	9.29 (2.17)	13.74 (2.08)	16.60 (2.01)	14.61 (2.01)	13.77 (2.06)	13.41 (2.08)	13.12 (2.07)
CC(1/2)‡	99.9 (71.4)	99.8 (78.8)	99.6 (91.0)	99.8 (74.0)	99.9 (69.7)	99.9 (69.0)	99.8 (74.3)	99.8 (74.3)	99.8 (79.6)

† Highest resolution shell in parentheses.

‡ Karplus & Diederichs (2012 ▸).
